# Formation of a macrocycle from di­chloro­dimethyl­silane and a pyridoxalimine Schiff base ligand

**DOI:** 10.1107/S2056989021010185

**Published:** 2021-10-13

**Authors:** Uwe Böhme, Anke Schwarzer, Betty Günther

**Affiliations:** a Institut für Anorganische Chemie, Technische Universität Bergakademie Freiberg, Leipziger Str. 29, 09599 Freiberg, Germany; b Institut für Organische Chemie, Technische Universität Bergakademie Freiberg, Leipziger Str. 29, 09599 Freiberg, Germany

**Keywords:** crystal structure, organosiloxane, pyridoxal, Schiff base, macrocycle

## Abstract

The reaction of di­chloro­dimethyl­silane with a polydentate Schiff base ligand derived from pyridoxal and 2-ethano­lamine yielded the macrocyclic silicon compound (8*E*,22*E*)-4,4,12,18,18,26-hexa­methyl-3,5,17,19-tetra­oxa-8,13,22,27-tetra­aza-4,18-disilatri­cyclo­[22.4.0.0^10,15^]octa­cosa-1(24),8,10,12,14,22,25,27-octa­ene-11,25-diol, C_24_H_36_N_4_O_6_Si_2_. The asymmetric unit contains the half macrocycle with an intra­molecular O—H⋯N hydrogen bond between the imine nitro­gen atom and a neighbouring oxygen atom. The crystal structure is dominated by C—H⋯O and C—H⋯π inter­actions, which form a high ordered mol­ecular network.

## Abstract 2

The reaction of di­chloro­dimethyl­silane with a polydentate Schiff base ligand derived from pyridoxal and 2-ethano­lamine yielded a macrocyclic silicon compound.

## Chemical context

The heterocyclic aldehyde pyridoxal is one of the active forms of vitamin B_6_. This vitamin is an essential cofactor to a large number of enzymes that catalyze many reactions of amino acids (Sykes *et al.*, 1991 ▸). The coordination chemistry of Schiff bases generated from amino acids and pyridoxal with trans­ition metal ions has been investigated intensive (Christensen, 1957 ▸; Long *et al.*, 1980 ▸; Dawes *et al.*, 1982 ▸; Walz *et al.*, 1983 ▸; Rao *et al.*, 1985 ▸; Astheimer *et al.*, 1985 ▸; Sykes *et al.*, 1991 ▸; Costa Pessoa *et al.*, 1999 ▸). We are working on silicon complexes with tridentate *O*,*N*,*O*-ligands (Böhme & Günther, 2007*a* ▸; Böhme *et al.*, 2006 ▸; Paul *et al.*, 2014 ▸; Warncke *et al.*, 2012 ▸; Schwarzer *et al.*, 2018 ▸). Therefore, we prepared a Schiff base from pyridoxal and 2-amino­ethanol as a potential *O*,*N*,*O*-ligand. The crystal structure of this mol­ecule, 4-[(2-hy­droxy­eth­yl)imino­meth­yl]-5- hy­droxy­methyl-2-methyl­pyridine-3-ol (I), was published earlier (Böhme & Günther, 2007*b* ▸). Compound (I) was used recently as ligand mol­ecule to coordinate copper and silver ions (Annaraj & Neelakantan, 2014 ▸, 2015 ▸). Herein we report the results of reaction between (I) and di­chloro­dimethyl­silane.

There are several potential coordination sites at the ligand mol­ecule (I): the pyridine and the imino nitro­gen atoms, two aliphatic and one phenolic hydroxyl groups. The presence of these functional groups makes it difficult to predict the structure of the reaction product with di­chloro­dimethyl­silane. It was our initial goal to prepare a penta­coordinate silicon complex like (II). Surprisingly the macrocyclic silicon compound (III) was obtained from the reaction of (I) with Me_2_SiCl_2_. The reaction was performed in tetra­hydro­furan in presence of triethylamine as supporting base to remove the hydrogen chloride, which is formed during the reaction. Recrystallization of the raw product from 1,2-di­meth­oxy­ethane and diethyl ether gave yellow crystals suitable for structure analysis. 
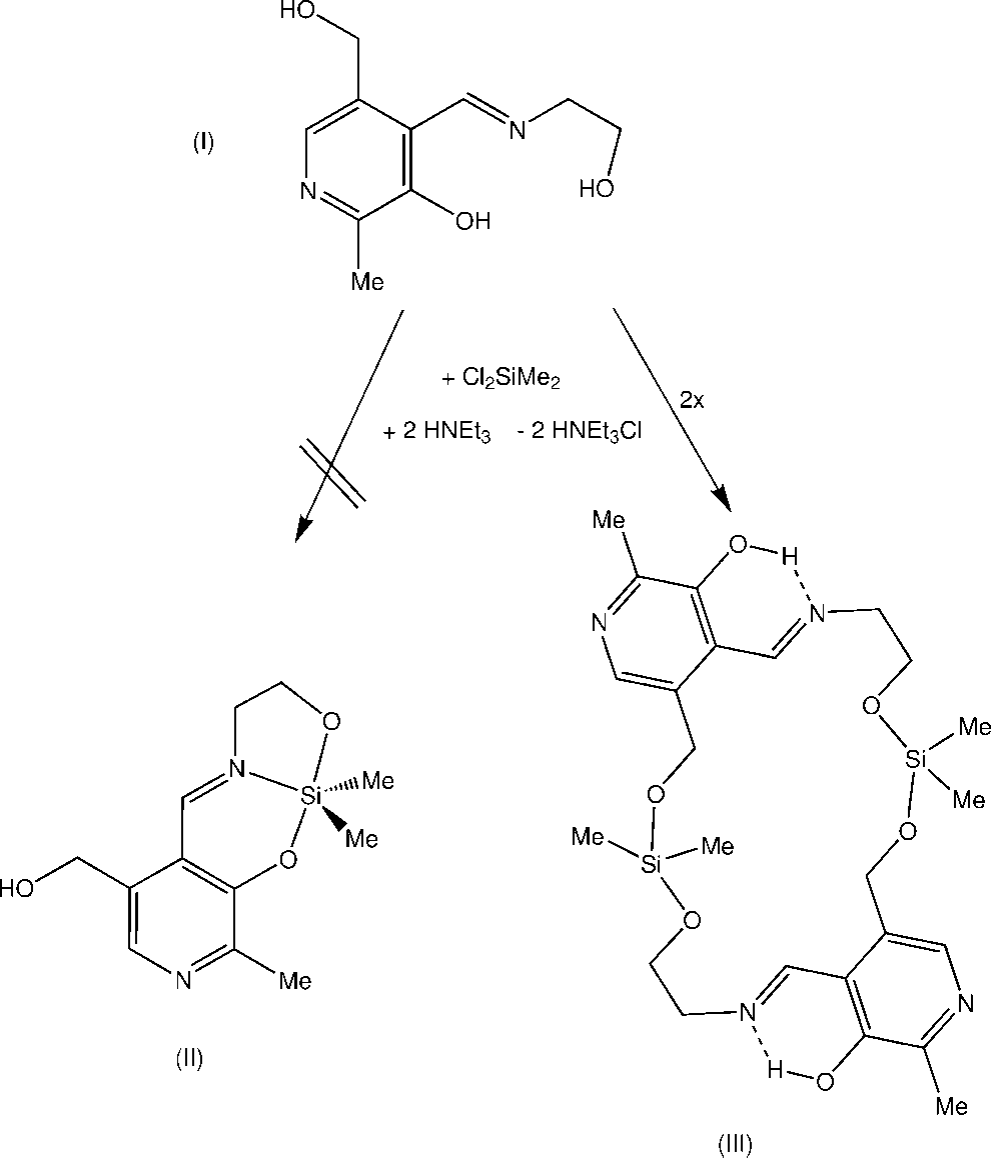


## Structural commentary

**Figure 1:**
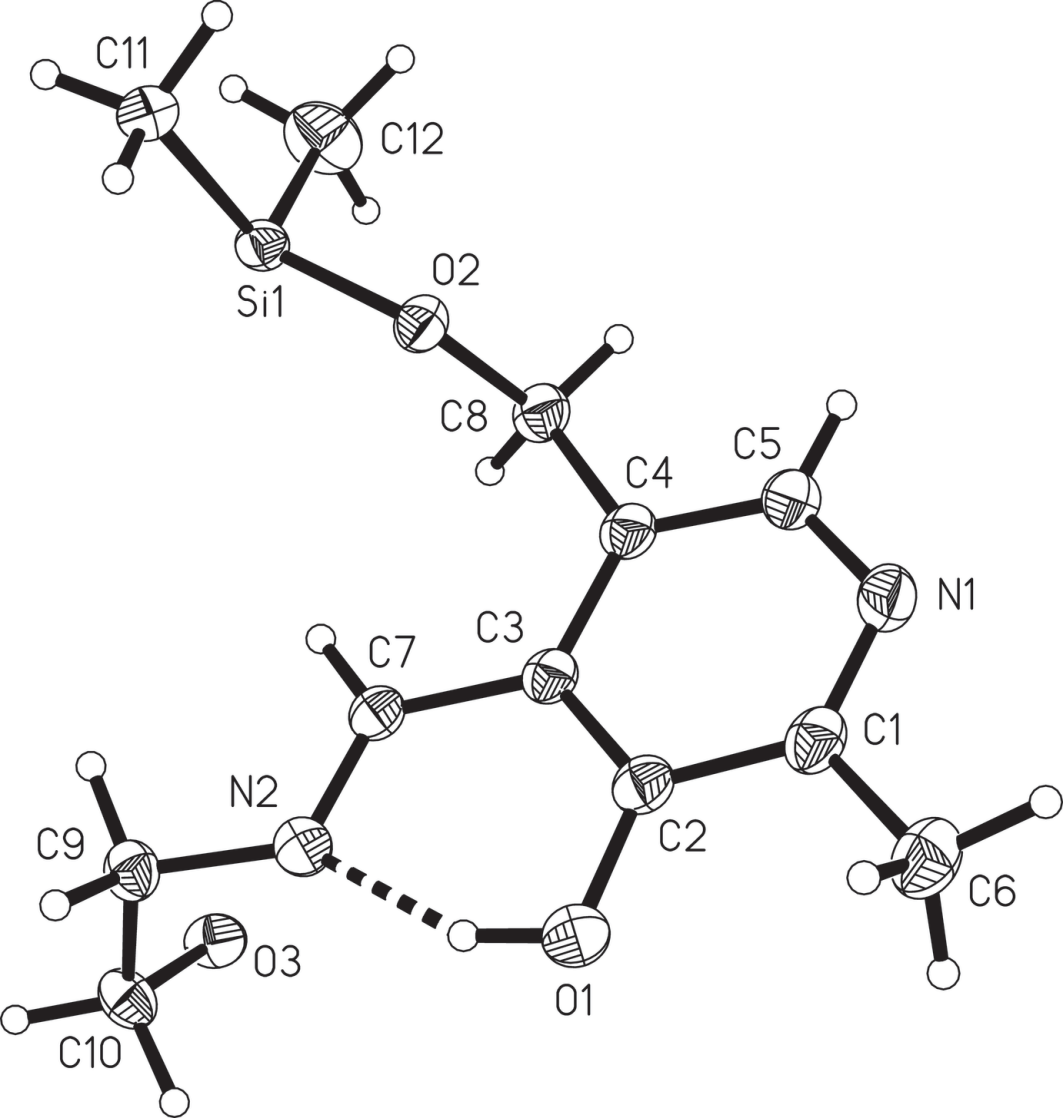
The asymmetric unit of (III), drawn with 50% probability displacement ellipsoids. The dashed line shows the intra­molecular O1—H1⋯N2 hydrogen bond.

**Figure 2:**
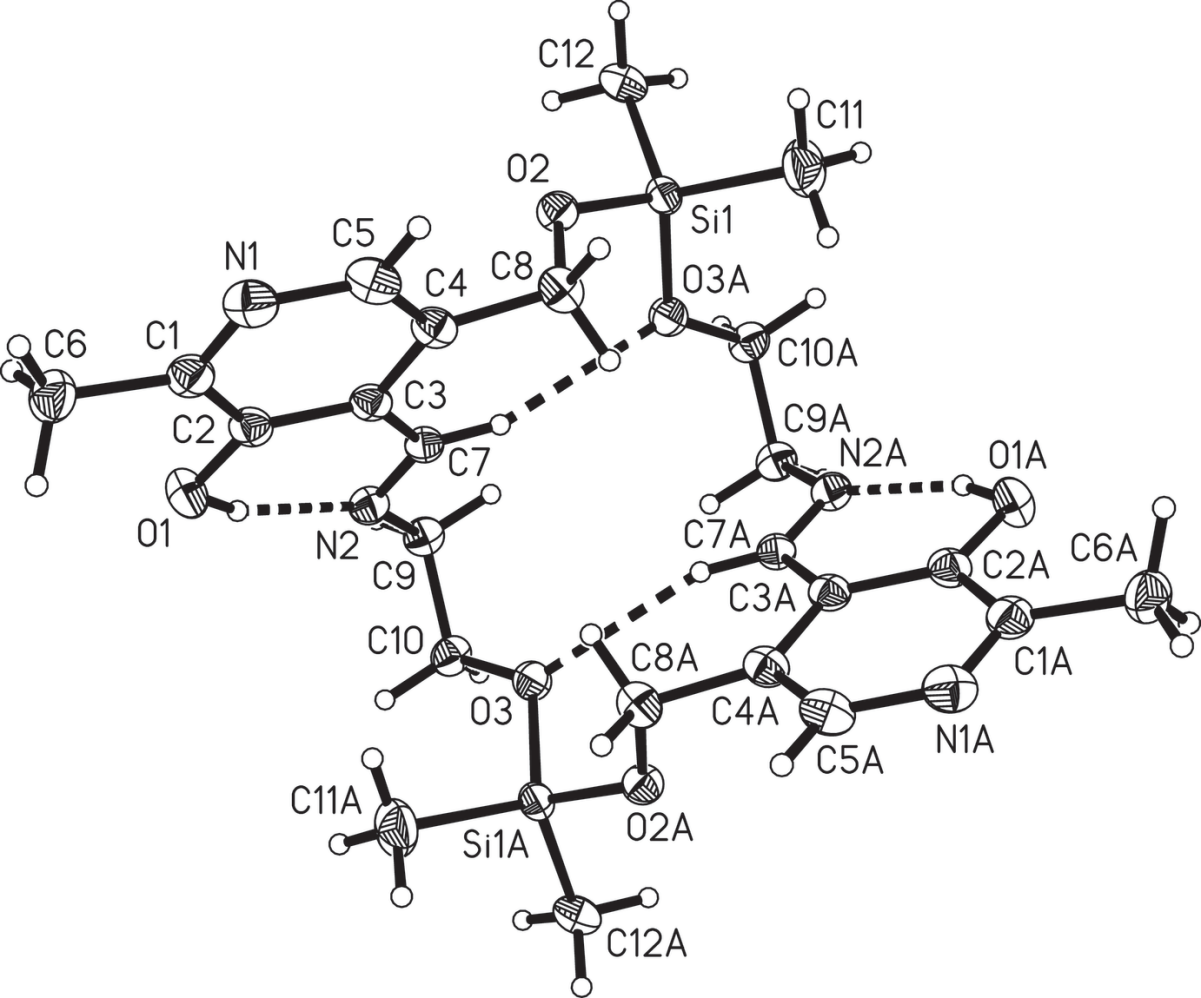
The mol­ecular structure of (III), drawn with 50% probability displacement ellipsoids.

Compound (III) crystallizes in the monoclinic space group *I*2/*c* with the half macrocycle in the asymmetric unit. Fig. 1 ▸ shows the asymmetric unit and the atomic labelling scheme. The macrocycle is generated by a crystallographic *C*2 axis through the centre of the macrocycle (Fig. 2 ▸). The silicon atom is bound to the two methyl groups and to the aliphatic oxygen atoms O2 and O3, thus forming a macrocycle (Fig. 2 ▸). A quite similar macrocycle has been obtained from the reaction of a related pyridoxal-derived Schiff base and di­chloro­diphenyl­silane (Böhme *et al.*, 2008 ▸). The short Si—O bonds (see Table 1 ▸) are in the range for comparable Si—O bonds (Wagler *et al.*, 2005 ▸; Böhme *et al.*, 2006 ▸, 2008 ▸; Böhme & Günther, 2007*a* ▸; Böhme & Foehn, 2007 ▸). The silicon atom is distorted tetra­hedral with bond angles between 103.40 (5) and 113.16 (7)° (Table 1 ▸). The rather large bond angles at the oxygen atoms (see Table 1 ▸) have been explained by the ionic character of the Si—O bonds (Gillespie & Johnson, 1997 ▸). There is a strong intra­molecular O—H⋯N inter­action (entry 1, Table 2 ▸) between the imine nitro­gen atom N2 and the O1—H1 group in the neighbouring position at the pyridoxal ring. The formation of hydrogen bridges between the imine nitro­gen atom and an *ortho*-hydroxyl group is a feature that is often observed in Schiff bases with *o*-hy­droxy groups (Hökelek *et al.*, 2004 ▸; Filarowski *et al.*, 1999 ▸). This strong intra­molecular O—H⋯N inter­action leads to a six-membered pseudo ring consisting of H1—O1—C2—C3—C7—N2. This pseudo ring is planar with an r.m.s. deviation of 0.009 Å from the ring plane. According to the graph-set notation proposed by Etter *et al.* (1990 ▸), these hydrogen bonds form motifs with an 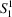 (6) graph-set descriptor. The hydrogen bonds C7—H7⋯O3 link different parts within one macrocycle *via* intra-annular hydrogen bonds (Fig. 2 ▸).

## Supra­molecular features

**Figure 3:**
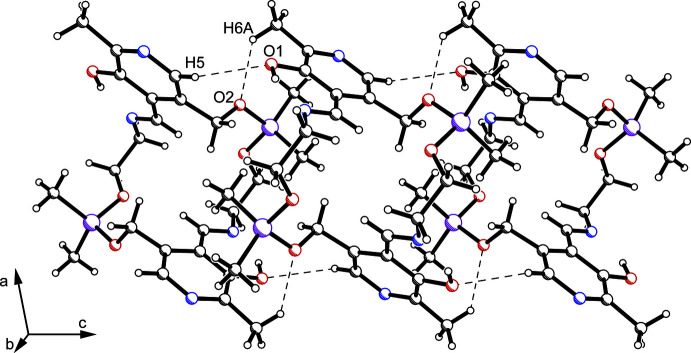
Packing excerpt of (III) showing C—H⋯O hydrogen bonds (dashed lines).

A bifurcated inter­molecular C—H⋯O inter­action is observed at O2 (Table 2 ▸). The inter­action of C6—H6*A*⋯O2 and C5—H5⋯O1 results in a chain along the crystallographic *b*-axis. The C—H⋯O inter­action of C9—H9*B* with O2 connects adjacent chains (Fig. 3 ▸).

Apart from the relevant C—H⋯O inter­action, two C—H⋯π contacts with the pyridine moiety (*Cg*1) are observed. First, a bifurcation at H9*B* (*d* = 3.31 Å) shows up within the C—H⋯O chains along the *c* axis. Furthermore, C11—H11*A*⋯*Cg*1 (*d* = 2.85 Å) supports the C—H⋯O inter­actions of H5 and H6*A*.

In summary, the crystal structure is dominated by C—H⋯O and C—H⋯π inter­actions, forming a highly ordered mol­ecular network.

The potential bonding sites in combination with the cavity of the macrocycle makes (III) a suitable candidate for supra­molecular recognition processes. The available pyridine N, azomethine N, and OH groups could be useful for the generation of nanostructures *via* complexation with transition metals (Leininger *et al.*, 2000 ▸).

## Database survey

A CSD search with *ConQuest* (Bruno *et al.*, 2002 ▸) for macrocycles containing Schiff bases from pyridoxal and 2-amino­alcohols showed that only one comparable silicon compound exists (Böhme *et al.*, 2008 ▸, refcode MOKVEO). The main differences between these two structures of silicon-containing macrocycles are as follows. First, (III) was found to crystallize without solvent while MOKVEO encloses chloro­fom mol­ecules. Probably as a result, the symmetry is lower in MOKVEO (triclinic, *P* 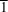 ) than in (III) showing the monoclinic *I*2/*c* symmetry. On the basis of the structure of (III) presented here and the former investigation (Böhme *et al.*, 2008 ▸), it can be assumed that pyridoxalimine-derived Schiff bases prefer the formation of macrocycles with diorganosilane units. However, it seems to be possible that compound (I) can also act as a tridentate *O*,*N*,*O*-ligand, as was shown recently with a hexa­coordinate titanium complex (Böhme & Günther, 2020 ▸).

## Synthesis and crystallization

The preparation of (III) was performed in Schlenk tubes under argon with dry and air-free solvents.

Compound (III) was prepared by reaction of 4-[(2-hy­droxy­eth­yl)imino­meth­yl]-5-hy­droxy­methyl-2-methyl­pyridine-3-ol (I) (1.7 g, 8 mmol) with di­chloro­dimethyl­silane (1.03 g, 8 mmol) in the presence of tri­ethyl­amine (2.02 g, 20 mmol). The reaction was performed in dry tetra­hydro­furan at room temperature. A white precipitate of tri­ethyl­amine hydro­chloride formed upon stirring of the mixture for five days. After this period, the tri­ethyl­amine hydro­chloride was filtered off and washed with tetra­hydro­furan. The solvent was removed *in vacuo* from the resulting clear yellow solution. The remaining solid was extracted with 1,2-di­meth­oxy­ethane. Addition of diethyl ether and cooling to 278 K yielded yellow crystals of (III) (1.66 g, 78%, m.p. 390 K).

NMR (CDCl_3_, 300 K, TMS, in p.p.m.): ^29^Si: −0.1. ^1^H: δ = 0.14 (_*s*_, Me_2_Si, 6H), 2.50 (*s*, CH_3_ pyridoxal, 3H), 3.71, 3.90 (*t*, N—CH_2_—CH_2_—O, 4H), 4.78 (*s*, CH_2_—O pyridoxal, 2H), 7.89 (*s*, CH pyridoxal, 1H), 8.84 (*s*, HC=N, 1H), 14.05 (*s*, OH pyridoxal, 1H). ^13^C: 3.0 (Me_2_Si), 22.0 (CH_3_ pyridoxal), 63.3, 64.6 (N—CH_2_—CH_2_—O), 66.4 (CH_2_—O pyridoxal), 122.6, 133.4, 140.8, 153.8, 157.8 (five C pyridoxal), 167.5 (HC=N).

## Refinement

Crystal data, data collection and structure refinement details are summarized in Table 3 ▸. The hydrogen atom at O1 was refined freely. The methyl groups were refined as idealized rigid groups allowed to rotate but not tip (AFIX 137; C—H = 0.98 Å, H—C—H = 109.5°). Other hydrogens were included using a riding model starting from calculated positions (C—H_aromatic_ = 0.95, C—H_methyl­ene_ = 0.99 Å). The *U*_iso_(H) values were fixed at 1.5 (for the methyl H) or 1.2 times the equivalent *U*_eq_ value of the parent carbon atoms.

## Supplementary Material

Crystal structure: contains datablock(s) I, I_7. DOI: 10.1107/S2056989021010185/zq2266sup1.cif

Structure factors: contains datablock(s) I. DOI: 10.1107/S2056989021010185/zq2266Isup2.hkl

Supporting information file. DOI: 10.1107/S2056989021010185/zq2266Isup3.cml

CCDC reference: 2113407

Additional supporting information:  crystallographic information; 3D view; checkCIF report

## Figures and Tables

**Table 1:** Selected geometric parameters (Å, °)

Si1—O2	1.6435 (9)	Si1—C12	1.8443 (14)
Si1—O3^i^	1.6487 (9)	Si1—C11	1.8589 (15)
			
O2—Si1—O3^i^	103.40 (5)	O3^i^—Si1—C11	109.52 (6)
O2—Si1—C12	106.94 (6)	C12—Si1—C11	113.16 (7)
O3^i^—Si1—C12	112.06 (6)	C8—O2—Si1	123.61 (8)
O2—Si1—C11	111.33 (7)	C10—O3—Si1^i^	123.50 (8)

Symmetry code: (i) -x+2, y, -z+{\script{1\over 2}}.

**Table 2:** Hydrogen-bond geometry (Å, °) *Cg*1 is the centroid of the N1/C1–C5 ring.

*D*—H⋯*A*	*D*—H	H⋯*A*	*D*⋯*A*	*D*—H⋯*A*
O1—H1⋯N2	0.90 (2)	1.76 (2)	2.5923 (15)	153.2 (18)
C5—H5⋯O1^ii^	0.95	2.69	3.5451 (16)	151
C6—H6*A*⋯O2^iii^	0.98	2.59	3.3464 (17)	134
C7—H7⋯O3^i^	0.95	2.57	3.4882 (15)	162
C9—H9*B*⋯O2^iv^	0.99	2.60	3.5087 (16)	153
C9—H9*B*⋯*Cg*1^iv^	0.99	3.31	4.039 (2)	131
C11—H11*A*⋯*Cg*1^ii^	0.98	2.85	3.7880 (2)	160

Symmetry codes: (i) -x+2, y, -z+{\script{1\over 2}}; (ii) x, -y+1, z-{\script{1\over 2}}; (iii) x, -y+1, z+{\script{1\over 2}}; (iv) -x+{\script{3\over 2}}, -y+{\script{1\over 2}}, -z+{\script{1\over 2}}.

**Table 3:** Experimental details

Crystal data	
Chemical formula	C_24_H_36_N_4_O_6_Si_2_
*M*_r_	532.75
Crystal system, space group	Monoclinic, *I*2/*c*
Temperature (K)	153
*a*, *b*, *c* (Å)	12.9641 (8), 16.8966 (7), 13.1085 (8)
β (°)	101.198 (5)
*V* (Å^3^)	2816.7 (3)
*Z*	4
Radiation type	Mo *K*α
μ (mm^−1^)	0.17
Crystal size (mm)	0.40 × 0.33 × 0.15

Data collection	
Diffractometer	Stoe IPDS 2T
Absorption correction	Integration (*X-RED*; Stoe, 2009 ▸)
*T*_min_, *T*_max_	0.907, 0.993
No. of measured, independent and observed [*I* > 2σ(*I*)] reflections	19293, 3242, 2833
*R*_int_	0.039
(sin θ/λ)_max_ (Å^−1^)	0.650

Refinement	
*R*[*F* ^2^ > 2σ(*F* ^2^)], *wR*(*F* ^2^), *S*	0.032, 0.082, 1.08
No. of reflections	3242
No. of parameters	169
H-atom treatment	H atoms treated by a mixture of independent and constrained refinement
Δρ_max_, Δρ_min_ (e Å^−3^)	0.32, −0.23

Computer programs: *X-AREA* and *X-RED* (Stoe, 2009 ▸), *SHELXS* (Sheldrick, 2008 ▸), *SHELXL2017/1* (Sheldrick, 2015 ▸) and *ORTEP-3 for Windows* (Farrugia, 2012 ▸).

## supplementary crystallographic information

### Crystal data

**Table:**

C_24_H_36_N_4_O_6_Si_2_	*F*(000) = 1136
*M**_r_* = 532.75	*D*_x_ = 1.256 Mg m^−^^3^
Monoclinic, *I*2/*c*	Mo *K*α radiation, λ = 0.71073 Å
*a* = 12.9641 (8) Å	Cell parameters from 19293 reflections
*b* = 16.8966 (7) Å	θ = 3.2–28.8°
*c* = 13.1085 (8) Å	µ = 0.17 mm^−^^1^
β = 101.198 (5)°	*T* = 153 K
*V* = 2816.7 (3) Å^3^	Prism, yellow
*Z* = 4	0.40 × 0.33 × 0.15 mm

### Data collection

**Table:**

Stoe IPDS 2T diffractometer	3242 independent reflections
Radiation source: sealed X-ray tube, 12 x 0.4 mm long-fine focus	2833 reflections with *I* > 2σ(*I*)
Plane graphite monochromator	*R*_int_ = 0.039
Detector resolution: 6.67 pixels mm^-1^	θ_max_ = 27.5°, θ_min_ = 2.0°
rotation method scans	*h* = −16→16
Absorption correction: integration (X-RED; Stoe, 2009)	*k* = −21→21
*T*_min_ = 0.907, *T*_max_ = 0.993	*l* = −16→16
19293 measured reflections	

### Refinement

**Table:**

Refinement on *F*^2^	0 restraints
Least-squares matrix: full	Hydrogen site location: mixed
*R*[*F*^2^ > 2σ(*F*^2^)] = 0.032	H atoms treated by a mixture of independent and constrained refinement
*wR*(*F*^2^) = 0.082	*w* = 1/[σ^2^(*F*_o_^2^) + (0.0343*P*)^2^ + 2.1897*P*] where *P* = (*F*_o_^2^ + 2*F*_c_^2^)/3
*S* = 1.08	(Δ/σ)_max_ = 0.001
3242 reflections	Δρ_max_ = 0.32 e Å^−^^3^
169 parameters	Δρ_min_ = −0.23 e Å^−^^3^

### Special details

**Table:**

Geometry. All esds (except the esd in the dihedral angle between two l.s. planes) are estimated using the full covariance matrix. The cell esds are taken into account individually in the estimation of esds in distances, angles and torsion angles; correlations between esds in cell parameters are only used when they are defined by crystal symmetry. An approximate (isotropic) treatment of cell esds is used for estimating esds involving l.s. planes.

### Fractional atomic coordinates and isotropic or equivalent isotropic displacement parameters (Å^2^)

**Table:**

	*x*	*y*	*z*	*U*_iso_*/*U*_eq_	
Si1	0.86455 (3)	0.32645 (2)	−0.02263 (2)	0.02075 (10)	
O1	0.71097 (8)	0.39251 (6)	0.42399 (7)	0.0296 (2)	
H1	0.7514 (16)	0.3499 (12)	0.4190 (14)	0.044*	
O2	0.80401 (7)	0.38781 (5)	0.04330 (7)	0.02281 (19)	
O3	1.06149 (7)	0.27321 (5)	0.43122 (7)	0.02520 (19)	
N1	0.67254 (9)	0.55915 (7)	0.24920 (9)	0.0294 (2)	
N2	0.83696 (8)	0.29351 (6)	0.35941 (8)	0.0230 (2)	
C1	0.66458 (10)	0.51009 (8)	0.32707 (10)	0.0259 (3)	
C2	0.72289 (10)	0.43890 (7)	0.34287 (9)	0.0233 (2)	
C3	0.78833 (9)	0.41735 (7)	0.27376 (9)	0.0209 (2)	
C4	0.79399 (10)	0.46943 (7)	0.19053 (9)	0.0229 (2)	
C5	0.73609 (11)	0.53867 (8)	0.18313 (10)	0.0282 (3)	
H5	0.741329	0.574080	0.128082	0.034*	
C6	0.58959 (11)	0.53139 (9)	0.39701 (11)	0.0338 (3)	
H6A	0.629045	0.540267	0.467780	0.051*	
H6B	0.539444	0.488047	0.397491	0.051*	
H6C	0.551402	0.579715	0.371352	0.051*	
C7	0.84345 (9)	0.34072 (7)	0.28447 (9)	0.0206 (2)	
H7	0.884566	0.326031	0.234897	0.025*	
C8	0.85625 (10)	0.45009 (7)	0.10775 (10)	0.0252 (3)	
H8A	0.861902	0.497621	0.065010	0.030*	
H8B	0.928130	0.433117	0.140566	0.030*	
C9	0.89177 (10)	0.21754 (7)	0.36341 (10)	0.0236 (2)	
H9A	0.914711	0.208345	0.296627	0.028*	
H9B	0.843029	0.174374	0.373348	0.028*	
C10	0.98674 (10)	0.21622 (7)	0.45164 (10)	0.0248 (3)	
H10A	0.964942	0.228849	0.518104	0.030*	
H10B	1.018780	0.162825	0.457622	0.030*	
C11	0.94958 (13)	0.38050 (10)	−0.09824 (13)	0.0406 (4)	
H11A	0.907504	0.419996	−0.142798	0.061*	
H11B	0.979557	0.342955	−0.141483	0.061*	
H11C	1.006491	0.407029	−0.050283	0.061*	
C12	0.76208 (11)	0.26635 (8)	−0.10486 (10)	0.0284 (3)	
H12A	0.717809	0.241394	−0.061306	0.043*	
H12B	0.795342	0.225328	−0.140318	0.043*	
H12C	0.718640	0.300404	−0.156636	0.043*	

### Atomic displacement parameters (Å^2^)

**Table:**

	*U*^11^	*U*^22^	*U*^33^	*U*^12^	*U*^13^	*U*^23^
Si1	0.02231 (17)	0.02191 (17)	0.01900 (16)	0.00084 (12)	0.00636 (12)	0.00114 (12)
O1	0.0360 (5)	0.0297 (5)	0.0259 (4)	0.0028 (4)	0.0128 (4)	0.0013 (4)
O2	0.0232 (4)	0.0221 (4)	0.0228 (4)	−0.0002 (3)	0.0034 (3)	−0.0036 (3)
O3	0.0241 (4)	0.0287 (5)	0.0230 (4)	−0.0076 (4)	0.0054 (3)	−0.0008 (3)
N1	0.0338 (6)	0.0237 (5)	0.0288 (5)	0.0025 (4)	0.0011 (4)	−0.0044 (4)
N2	0.0219 (5)	0.0229 (5)	0.0237 (5)	−0.0010 (4)	0.0029 (4)	0.0000 (4)
C1	0.0251 (6)	0.0259 (6)	0.0254 (6)	−0.0010 (5)	0.0012 (5)	−0.0074 (5)
C2	0.0240 (6)	0.0240 (6)	0.0209 (5)	−0.0033 (5)	0.0020 (4)	−0.0039 (4)
C3	0.0214 (5)	0.0206 (5)	0.0195 (5)	−0.0040 (4)	0.0011 (4)	−0.0031 (4)
C4	0.0264 (6)	0.0203 (6)	0.0211 (5)	−0.0049 (4)	0.0026 (4)	−0.0033 (4)
C5	0.0374 (7)	0.0213 (6)	0.0246 (6)	−0.0017 (5)	0.0026 (5)	−0.0019 (5)
C6	0.0283 (7)	0.0370 (7)	0.0363 (7)	0.0046 (6)	0.0072 (5)	−0.0080 (6)
C7	0.0200 (5)	0.0218 (6)	0.0196 (5)	−0.0030 (4)	0.0024 (4)	−0.0033 (4)
C8	0.0306 (6)	0.0210 (6)	0.0246 (6)	−0.0058 (5)	0.0071 (5)	−0.0014 (4)
C9	0.0237 (6)	0.0203 (6)	0.0263 (6)	−0.0027 (4)	0.0034 (5)	−0.0003 (4)
C10	0.0229 (6)	0.0231 (6)	0.0280 (6)	−0.0032 (5)	0.0040 (5)	0.0049 (5)
C11	0.0394 (8)	0.0453 (9)	0.0415 (8)	−0.0018 (7)	0.0192 (7)	0.0115 (7)
C12	0.0370 (7)	0.0264 (6)	0.0204 (6)	−0.0002 (5)	0.0024 (5)	−0.0014 (5)

### Geometric parameters (Å, º)

**Table:**

Si1—O2	1.6435 (9)	C5—H5	0.9500
Si1—O3^i^	1.6487 (9)	C6—H6A	0.9800
Si1—C12	1.8443 (14)	C6—H6B	0.9800
Si1—C11	1.8589 (15)	C6—H6C	0.9800
O1—C2	1.3539 (15)	C7—H7	0.9500
O1—H1	0.90 (2)	C8—H8A	0.9900
O2—C8	1.4345 (14)	C8—H8B	0.9900
O3—C10	1.4278 (14)	C9—C10	1.5168 (17)
N1—C1	1.3343 (18)	C9—H9A	0.9900
N1—C5	1.3512 (18)	C9—H9B	0.9900
N2—C7	1.2808 (16)	C10—H10A	0.9900
N2—C9	1.4631 (16)	C10—H10B	0.9900
C1—C2	1.4143 (18)	C11—H11A	0.9800
C1—C6	1.5039 (18)	C11—H11B	0.9800
C2—C3	1.4041 (17)	C11—H11C	0.9800
C3—C4	1.4147 (17)	C12—H12A	0.9800
C3—C7	1.4723 (17)	C12—H12B	0.9800
C4—C5	1.3832 (18)	C12—H12C	0.9800
C4—C8	1.5085 (17)		
			
O2—Si1—O3^i^	103.40 (5)	N2—C7—H7	119.4
O2—Si1—C12	106.94 (6)	C3—C7—H7	119.4
O3^i^—Si1—C12	112.06 (6)	O2—C8—C4	108.98 (10)
O2—Si1—C11	111.33 (7)	O2—C8—H8A	109.9
O3^i^—Si1—C11	109.52 (6)	C4—C8—H8A	109.9
C12—Si1—C11	113.16 (7)	O2—C8—H8B	109.9
C2—O1—H1	104.4 (12)	C4—C8—H8B	109.9
C8—O2—Si1	123.61 (8)	H8A—C8—H8B	108.3
C10—O3—Si1^i^	123.50 (8)	N2—C9—C10	110.90 (10)
C1—N1—C5	118.59 (11)	N2—C9—H9A	109.5
C7—N2—C9	118.05 (11)	C10—C9—H9A	109.5
N1—C1—C2	121.46 (12)	N2—C9—H9B	109.5
N1—C1—C6	118.23 (12)	C10—C9—H9B	109.5
C2—C1—C6	120.30 (12)	H9A—C9—H9B	108.0
O1—C2—C3	122.14 (11)	O3—C10—C9	109.10 (10)
O1—C2—C1	117.91 (11)	O3—C10—H10A	109.9
C3—C2—C1	119.92 (11)	C9—C10—H10A	109.9
C2—C3—C4	117.69 (11)	O3—C10—H10B	109.9
C2—C3—C7	120.63 (11)	C9—C10—H10B	109.9
C4—C3—C7	121.57 (11)	H10A—C10—H10B	108.3
C5—C4—C3	118.12 (12)	Si1—C11—H11A	109.5
C5—C4—C8	119.45 (11)	Si1—C11—H11B	109.5
C3—C4—C8	122.36 (11)	H11A—C11—H11B	109.5
N1—C5—C4	124.19 (12)	Si1—C11—H11C	109.5
N1—C5—H5	117.9	H11A—C11—H11C	109.5
C4—C5—H5	117.9	H11B—C11—H11C	109.5
C1—C6—H6A	109.5	Si1—C12—H12A	109.5
C1—C6—H6B	109.5	Si1—C12—H12B	109.5
H6A—C6—H6B	109.5	H12A—C12—H12B	109.5
C1—C6—H6C	109.5	Si1—C12—H12C	109.5
H6A—C6—H6C	109.5	H12A—C12—H12C	109.5
H6B—C6—H6C	109.5	H12B—C12—H12C	109.5
N2—C7—C3	121.19 (11)		
			
O3^i^—Si1—O2—C8	68.47 (10)	C2—C3—C4—C8	−176.10 (11)
C12—Si1—O2—C8	−173.10 (9)	C7—C3—C4—C8	0.08 (17)
C11—Si1—O2—C8	−49.03 (11)	C1—N1—C5—C4	0.17 (19)
C5—N1—C1—C2	1.61 (18)	C3—C4—C5—N1	−1.42 (19)
C5—N1—C1—C6	−176.99 (12)	C8—C4—C5—N1	175.64 (12)
N1—C1—C2—O1	179.60 (11)	C9—N2—C7—C3	178.48 (10)
C6—C1—C2—O1	−1.83 (17)	C2—C3—C7—N2	−3.14 (17)
N1—C1—C2—C3	−2.09 (18)	C4—C3—C7—N2	−179.21 (11)
C6—C1—C2—C3	176.48 (11)	Si1—O2—C8—C4	−154.64 (8)
O1—C2—C3—C4	179.01 (11)	C5—C4—C8—O2	−106.34 (13)
C1—C2—C3—C4	0.78 (17)	C3—C4—C8—O2	70.59 (14)
O1—C2—C3—C7	2.80 (17)	C7—N2—C9—C10	108.51 (12)
C1—C2—C3—C7	−175.43 (10)	Si1^i^—O3—C10—C9	143.92 (9)
C2—C3—C4—C5	0.87 (16)	N2—C9—C10—O3	−65.04 (13)
C7—C3—C4—C5	177.05 (11)		

Symmetry code: (i) −*x*+2, *y*, −*z*+1/2.

### Hydrogen-bond geometry (Å, º)

*Cg*1 is the centroid of the N1/C1–C5 ring.

**Table:**

*D*—H···*A*	*D*—H	H···*A*	*D*···*A*	*D*—H···*A*
O1—H1···N2	0.90 (2)	1.76 (2)	2.5923 (15)	153.2 (18)
C5—H5···O1^ii^	0.95	2.69	3.5451 (16)	151
C6—H6*A*···O2^iii^	0.98	2.59	3.3464 (17)	134
C7—H7···O3^i^	0.95	2.57	3.4882 (15)	162
C9—H9*B*···O2^iv^	0.99	2.60	3.5087 (16)	153
C9—H9*B*···*Cg*1^iv^	0.99	3.31	4.039 (2)	131
C11—H11*A*···*Cg*1^ii^	0.98	2.85	3.7880 (2)	160

Symmetry codes: (i) −*x*+2, *y*, −*z*+1/2; (ii) *x*, −*y*+1, *z*−1/2; (iii) *x*, −*y*+1, *z*+1/2; (iv) −*x*+3/2, −*y*+1/2, −*z*+1/2.
